# Heat pump supply chain environmental impact reduction to improve the UK energy sustainability, resiliency and security

**DOI:** 10.1038/s41598-023-47850-x

**Published:** 2023-11-23

**Authors:** Moein Shamoushaki, S. C. Lenny Koh

**Affiliations:** 1 Sheffield University Management School, The University of Sheffield, Sheffield, S10 1FL UK; 2 Energy Institute, The University of Sheffield, Sheffield, S10 2TN UK

**Keywords:** Renewable energy, Environmental sciences, Energy infrastructure

## Abstract

Various heat pump technologies are examined from an environmental perspective using a life cycle assessment approach. The investigated heat pump systems utilize air, ground, and water as their energy sources. Additionally, an innovative heat pump powered by green hydrogen is investigated in this study, to evaluate its environmental impacts and potential to commercialise on a large scale. A range of supply chain scenarios is explored, considering the main suppliers of the UK market. The reshoring heat pump industry and supply chain are evaluated to enhance energy resilience and security within the UK. The findings indicate that the hydrogen-based heat pump presents a promising option for the UK market, offering the advantages of reducing stress on the national grid network and minimizing the environmental impacts associated with the supply chain. Furthermore, a forecasting analysis is conducted based on the UK's net-zero emission plan to provide insight into future developments.

## Introduction

The transition to low-carbon technologies to provide heating and cooling energy required for domestic and industry sectors and reduce the dependency on fossil fuels are among the top priorities of the governments. However, expanding certain technologies on a large scale should be aligned with climate change and greenhouse gas (GHG) emission reduction policies. The swift transition to renewable energy is anticipated to promote economic sustainability and emissions reduction, aligning with the principles of the European Green Deal^1^. Currently, numerous researchers specializing in transitions are addressing major sustainability issues like climate change^2^. Before bringing a technology to the market, it is crucial to evaluate its environmental impact. Newly conducted studies suggest that by 2050, there is an expected rise of 50% in the release of GHGs, establishing it as the main and most significant element driving the progression of climate change^3^. Taking sufficient preventive actions is crucial to prevent or alleviate the harm resulting from climate change^4^. Heat pump technologies represent ecologically conscious methods for enhancing energy efficiency, possessing the capability to substantially lower carbon emissions linked to the heating of buildings on a considerable magnitude^5^. A heat pump serves as an energy-conscious solution that can aid in regulating electricity consumption through intelligent demand-side management, particularly as renewable energy becomes more prevalent in the grid^6^. Several studies have been done on life cycle assessment (LCA) of different types of heat pump technologies.

The dominant heating system utilized in residential structures within the United Kingdom (UK) consists of central heating operated by a natural gas-fuelled boiler, accounting for approximately 92% in 2017^7^. Due to the implementation of the Future Homes Standard, the UK Government has set a nationwide goal to eliminate fossil fuel-based heating systems from new residential constructions beginning in 2025^8^. These pieces of evidence substantiate the notable need for increasingly eco-friendly technologies such as heat pumps in the near future. In the UK, the predominant portion of units sold, comprising 87%, consists of air-source heat pumps (ASHPs), trailed by ground-source heat pumps (GSHPs) and water-source heat pumps (WSHPs) at 9%. The residual 4% of the market is represented by hybrid systems, amalgamating a heat pump with a conventional fossil-fuel boiler in a compact configuration^9^. Outlined within the Heat and Buildings Strategy^10^, the UK Government detailed its intentions to achieve a minimum of 600,000 heat pump installations annually by 2028. This strategy also acknowledges the potential contribution of hydrogen in the decarbonization of heat, with the notion that hydrogen could cater to up to 4 million households by 2035^11^. The most promising opportunity for heat pumps in the UK lies within larger residences, particularly those that are not connected to the main power grid^12^. The benefits of hydrogen include its abundant availability, minimal emissions, a significant reduction in GHGs, and the reversibility of its generation process ^13^.

Given that a significant portion of heat pump components are imported from abroad, mitigating the environmental effects of the heat pump supply chain becomes imperative. To meet the net-zero emission target, emissions from heat and the building sector are required to be net-zero^14^. Numerous studies have focused on either ASHP^15^ or GSHP^16–19^, while others have conducted a comparative LCA of diverse heating and cooling systems (encompassing boilers, and hybrid units) and comparison with most conventional heat pump technologies such as ASHP and GSHP^20–28^. However, the water-source heat pump has received comparatively less attention in research endeavours^29^.

The UK's net-zero emission strategy concerning the building sector will necessitate the widespread adoption of low-carbon technologies such as heat pumps, which offer a viable replacement for gas-based boilers. Consequently, the heat pump market and its supply chain will play a significant role in shaping the energy security and resilience of the UK. Incorporating energy resilience into policy and program development can protect and expedite the shift toward accessible and cost-effective renewable energy for different sectors^30^. Increasing environmental and societal stresses resulting from possible disruptions in supply chains are compelling businesses to adopt sustainable and adaptable strategies^31^. Furthermore, adopting efficient and reliable low-carbon technologies can create a more resilient energy system, less vulnerable to market fluctuations, and enhance energy security and resilience^32^.

The majority of existing research has concentrated on assessing the environmental effects of three conventional heat pump technologies (ASHP, GSHP, and WSHP), however, in this study, we have assessed a novel heat pump type named hydrogen-based heat pump (HSHP) which operates using green hydrogen derived from renewable energy sources instead of relying on electricity from the grid network. In one research^28^, the possibility of utilizing hydrogen as an energy source for gas-powered equipment was explored. However, to the best of our knowledge, considering hydrogen as an energy source for heat pumps has not been studied before. Furthermore, an extensive set of supply chain scenarios has been expanded for all these technologies, drawing from the latest report released by the UK government and the main supplier within the UK market. The aim is to compare each scenario in terms of their environmental impacts and their connections with energy security and resiliency. A forecasting evaluation to find the damaging environmental impact reduction of the heat pump supply chain, aligned with the UK's net-zero emission plan by 2050. Additionally, the discussion extends to the influence of heat pump technology on energy resilience and security within the UK.

### Developed supply chain scenarios

**Figure 1:**
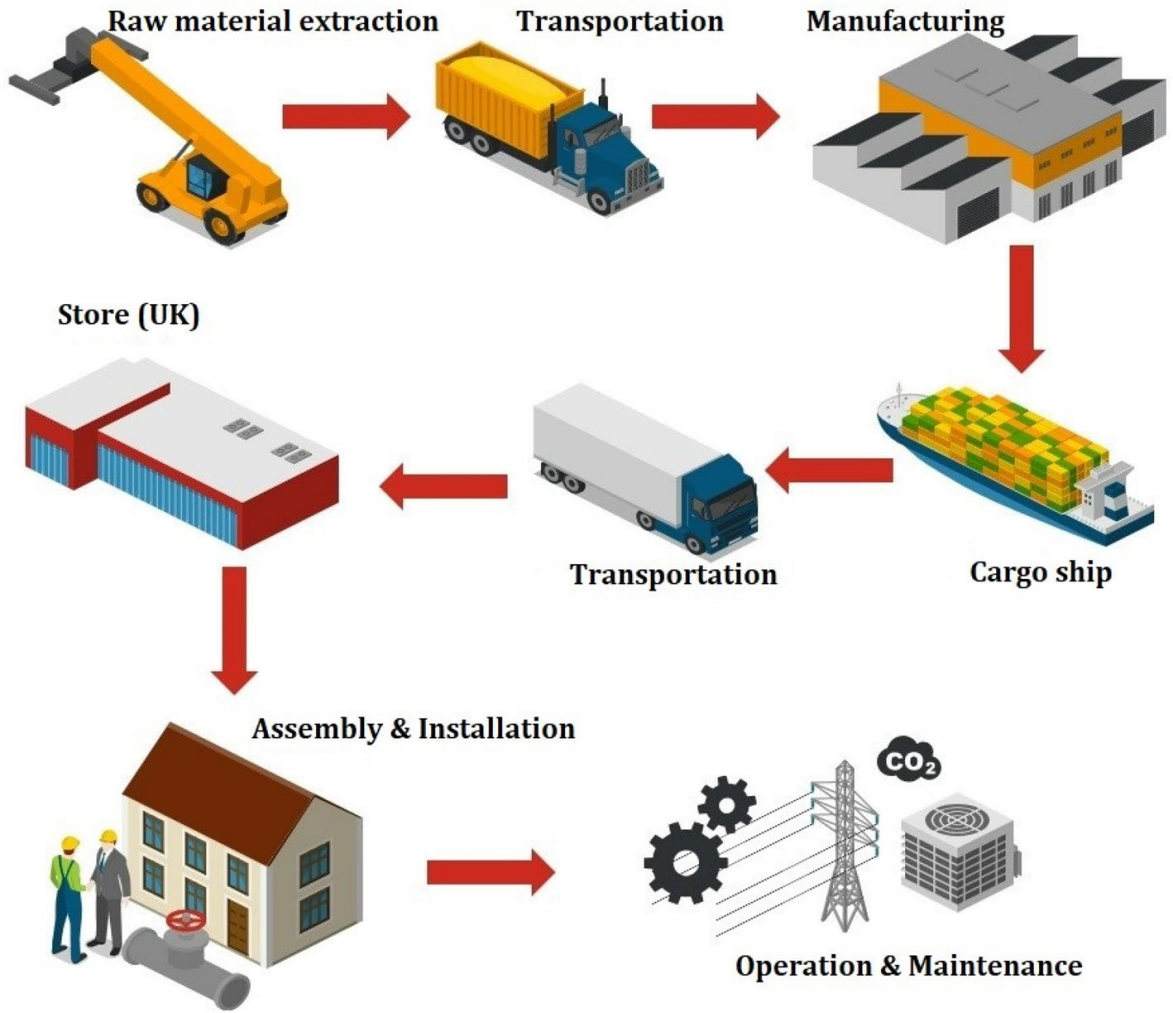
Simplified scheme of analysed heat pump supply chain, This study involves analysing various supply chain situations. It commences with the extraction of raw materials and their transportation to the manufacturing facility. Subsequently, a variety of transportation strategies, encompassing both cargo ships and trucks, are implemented to store products in a warehouse located in London. The study also takes into account aspects such as analysis and improvement of A&I as well as O&M.

**Figure 2:**
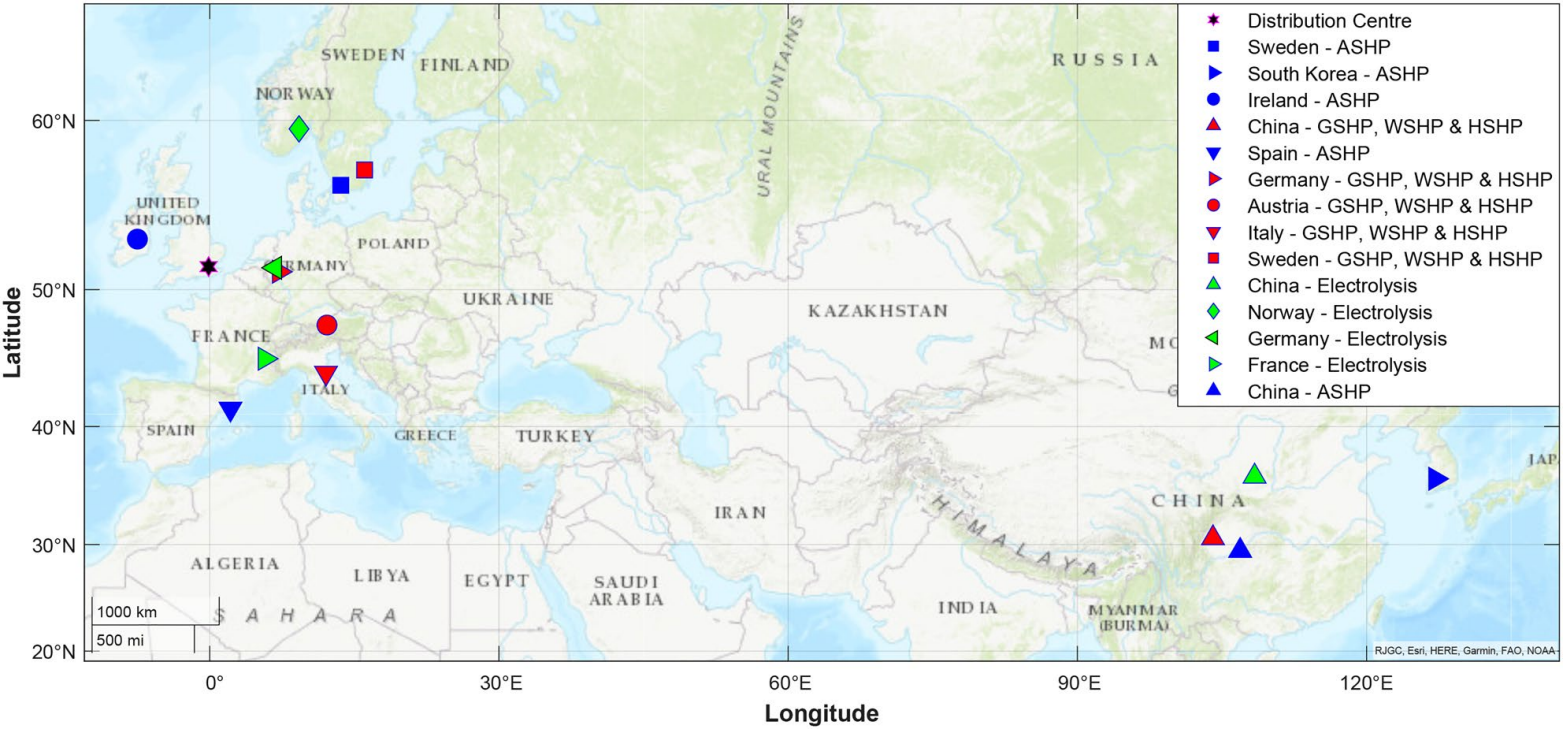
Heat pump and electrolysis components supplier mapping, The location of the manufacturer of components, and the start point of distribution are shown for all considered technologies. The final distribution centre is London which has been shown by a star on the map.

A sustainable and resilient supply chain is essential for improving the energy security of the building sector. The supply chain scenarios in this study are developed based on the most updated UK government report about the heat pump supply chain in the UK. The considered phases in this assessment are raw material extraction, transportation, manufacturing, Operation and Maintenance (O & M), Assembly and Installation (A & I). The simplified scheme of the general heat pump supply chain scheme is shown in Fig. 1. The final point of distribution is supposed to be the London store. The distribution to retailers and end-users is excluded from this study. The raw material extraction phase encompasses the entirety of the manufacturing process, including material extraction, related processes, and the manufacturing of components. Additionally, transportation aspects are integrated into the distribution phase to assess their influence on the overall life cycle of the supply chain. The location and countries of manufacturing heat pumps and electrolysis are shown in Fig. 2. Two main suppliers are China and South Korea in Asia, and others are from different parts of Europe. Several base scenarios are expanded considering 100% of the production of heat pump components in the exporter country (scenarios numbered from 1 to 5). Other scenarios are developed supposing a reshoring approach by establishing new factories in the UK or promoting available production capacity in the UK to supply 50% of heat pump equipment manufacturing in the UK and the remaining 50% importing from exporter country (scenarios numbered from 6 to 10). Another single scenario is assumed based on the 100% of heat pump components construction in the UK (scenarios numbered 11). The main purpose of this study is to compare different supply chain scenarios to figure out a sustainable and resilient heat pump supply chain for the UK. Scenarios numbered 12 to 14 are based on a predictive analysis of the environmental impact reduction of the heat pump supply chain in the UK aligned with the net-zero emission plan. The studied and analysed scenarios are summarised in Table 1.

**Table 1:** Developed scenarios for all technologies, The scenario for each technology is named with a letter and a number. 14 supply chain scenarios are expanded for all technology which means 56 scenarios are studied and compared in this research.

ASHP				GSHP					
Scenario	Manufacturing country	Manufacturing percentage (%)		Scenario	Manufacturing country	Manufacturing percentage (%)			
		Exporter	UK			Exporter		UK	
A1	Sweden	100	0	G1	Sweden	100		0	
A2	South Korea	100	0	G2	Germany	100		0	
A3	Ireland	100	0	G3	Austria	100		0	
A4	China	100	0	G4	Italy	100		0	
A5	Spain	100	0	G5	China	100		0	
A6	Sweden & UK	50	50	G6	Sweden & UK	50		50	
A7	South Korea & UK	50	50	G7	Germany & UK	50		50	
A8	Ireland & UK	50	50	G8	Austria & UK	50		50	
A9	China & UK	50	50	G9	Italy & UK	50		50	
A10	Spain & UK	50	50	G10	China & UK	50		50	
A11	UK (2023)	0	100	G11	UK (2023)	0		100	
A12	UK (2030)	0	100	G12	UK (2030)	0		100	
A13	UK (2040)	0	100	G13	UK (2040)	0		100	
A14	UK (2050)	0	100	G14	UK (2050)	0		100	

WSHP				HSHP					
Scenario	Manufacturing country	Manufacturing percentage (%)		Scenario	Manufacturing country	Manufacturing percentage (%)			
		Exporter	UK			Exporter		UK	
						Heat pump	electrolysis	Heat pump	electrolysis
W1	Sweden	100	0	H1	Sweden & France	100	100	0	0
W2	Germany	100	0	H2	Germany	100	100	0	0
W3	Austria	100	0	H3	Austria & France	100	100	0	0
W4	Italy	100	0	H4	Italy & France	100	100	0	0
W5	China	100	0	H5	China	100	100	0	0
W6	Sweden & UK	50	50	H6	Sweden, France & UK	50	50	50	50
W7	Germany & UK	50	50	H7	Germany & UK	50	50	50	50
W8	Austria & UK	50	50	H8	Austria, France & UK	50	50	50	50
W9	Italy & UK	50	50	H9	Italy, France & UK	50	50	50	50
W10	China & UK	50	50	H10	China & UK	50	50	50	50
W11	UK (2023)	0	100	H11	UK	0	0	100	100
W12	UK (2030)	0	100	H12	UK (2030)	0	0	100	100
W13	UK (2040)	0	100	H13	UK (2040)	0	0	100	100
W14	UK (2050)	0	100	H14	UK (2050)	0	0	100	100

## Methods

### Life cycle assessment

LCA emerges as a valuable instrument for making knowledgeable choices and systematically tracking a wide array of ecological effects across the complete spectrum of product creation. The utilization of LCA establishes and tackles crucial ecological sustainability concerns vital for subsequent growth and expansion^33^. Since the turn of the century, LCA has been put into practice through implementation in world policy^34^. The recognition of LCA's worth in quantitatively evaluating the environmental impacts of product life cycles has grown, and it is increasingly employed to aid decision-making in policy and governmental contexts^35^. All exchanges of energy and materials with the environment during the entire lifecycle should be gathered and analysed in terms of their ecological consequences^36^. The significance of LCAs lies in their capacity to reveal both the locations and the comparative significance of environmental effects^37^. It is a widely recognized and globally standardized approach employed to compute the environmental discharges associated with a product or process throughout its various life cycle stages^38^. The LCA carried out in this research adheres to the methodology outlined in the ISO 14,040 standard^39^. It comprises several main steps which are goal and scope definition, life cycle inventory analysis, impact assessment methodology, and interpretation of the results.

### Goal and scope definition

The primary step of the LCA study is determining the goal and scope of the project. The main goal of this study is to conduct a comparative life cycle supply chain assessment of different heat pump technologies including a novel system named HSHP. Diverse supply chain pathways are developed to compare carbon footprint. The supposed system boundary is cradle-to-gate. The selected functional unit for all systems is considered to be 1 kWh of generated energy. OpenLCA v. 1.11.0 software is applied to conduct the LCA modelling.

### Life cycle inventory analysis

In the process of inventory analysis, information is gathered concerning multiple elements, encompassing the utilization of resources, energy expenditure, water usage, emissions released into the air, water, and soil, as well as the creation of waste. The inventory analysis facilitates a thorough evaluation of the environmental effects of the material, allowing scholars to pinpoint and comprehend the critical environmental hotspot issues. This pinpointing of key areas can direct the focus of endeavours toward environmental enhancement and offer insights for decision-making. This stage establishes the groundwork for following LCA stages, which are impact assessment and interpretation^40^. Several databases are applied to collect the required data for systems modelling such as Environmental Footprint (EF), Ecoinvent v3.9 databases, and some literature^28,41,42^.

### Impact assessment method

In the life cycle impact assessment stage, characterization criteria are predominantly employed to convert the LCI data into quantitative estimations of the potential environmental consequences linked to the product^43^. ReCiPe 2016 midpoint method is applied to assess the environmental consequences of the systems in order to provide more detailed understanding of the underlying processes and help identify areas where more targeted improvements in reducing environmental impacts. While endpoint methods can provide a broader perspective, the midpoint approach aligns better with the research goals and provides the necessary granularity for the analysis.

### Interpretation

After conducting the assessment of environmental impacts, the outcomes need to be scrutinized and explained with the earlier investigations interpretation phase, which represents the final stage of LCA^44^. The interpretive stage entails a thorough evaluation of the outcomes of an LCA investigation, encompassing both the inventory and the impact assessment phases, aligning with the study's defined objectives and scope^45^.

## Results

**Figure 3:**
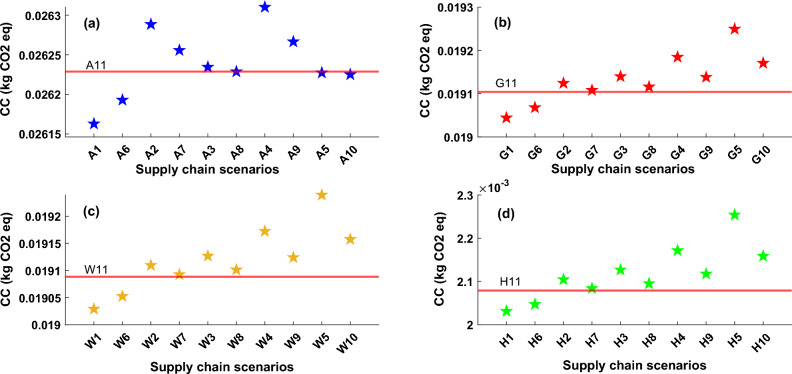
Climate change impact for all heat pump technologies, (**a**) ASHP, (**b**) GSHP, (**c**) WSHP, (**d**) HSHP, The values of climate change impact for all defined scenarios and technologies are presented on these graphs with star markers, red line in each plot represents the impact value based on the UK supply chain scenarios which named A11, G11, W11, and H11 for ASHP, GSHP, WSHP and HSHP types, respectively.

Here, the outcomes of environmental evaluation concerning some main impact categories stemming from the supply chain of the heat pump industry are presented. These impacts are selected as they have the most dominant damaging impacts. Figure 3 illustrates the supply chain climate change (CC) impact of all considered scenarios for all technologies. The red line in these graphs represents the UK Scenarios based on 2023 data. It should be mentioned that each supply chain scenario includes raw material extraction, transportation, manufacturing, A&I, and O&M. Results show that for all studied technologies, supply chain carbon dioxide emissions based on both 50% and 100% of manufacturing in Sweden have a lower damaging impact compared with other scenarios and the UK which is mainly due to lower pollution related to the energy mix in Sweden. Because renewable-based power generation is the majority portion of the energy mix in this country. Supply chain scenarios of manufacturing and importing from China is the most polluting solution due to higher fossil fuel-based energy in power generation in China which causes high pollution during the manufacturing process. These findings demonstrate the significant influence of the energy grid on the environmental consequences of the production process. Moreover, the extraction of materials and relevant processes in each region can differ based on factors such as the presence of pollutants, specific criteria, the availability of resources, energy requirements, varying processes, and logistical situations and types related to the production location. These are additional influential factors in the manufacturing aspect of supply chain scenarios.

Generally, the supply chain of ASHP exhibits a greater carbon footprint impact when compared to other technologies that have been examined. This discrepancy is primarily attributed to the materials employed during the manufacturing and O&M phases. The materials used in the construction of ASHP systems, such as low-alloyed and reinforcing steel, copper, and insulation materials for piping, exceed those used in other technologies. These specific materials are the primary drivers of the increased carbon footprint associated with the manufacturing process of ASHP. Furthermore, GSHP and WSHP necessitate a lesser quantity of these materials during the manufacturing stage. Another significant factor contributing to the higher carbon footprint of ASHP systems is the increased utilization of refrigerants in comparison to other cases. The UK scenario for ASHP supply chain carbon footprint is similar to supplying from Spain and Ireland. However, in other cases, the supply chain carbon footprint is lower for the UK scenarios (G11, W11, and H11). The most pollutant scenario for all studied cases, is China (both 50% and 100% of manufacturing capacity). Assuming 50% of manufacturing in the UK caused a decrease in carbon dioxide emission for all scenarios that will lead to less transportation-related pollution. It is evident that the carbon footprint of HSHP is consistently much lower than that of other technologies in all scenarios and supply chain pathways. This is primarily attributed to the elimination of electricity requirements for the system's operation throughout its lifespan. The reduction or elimination of reliance on the grid network has a beneficial effect on reducing carbon emissions during the operation of these systems.

**Figure 4:**
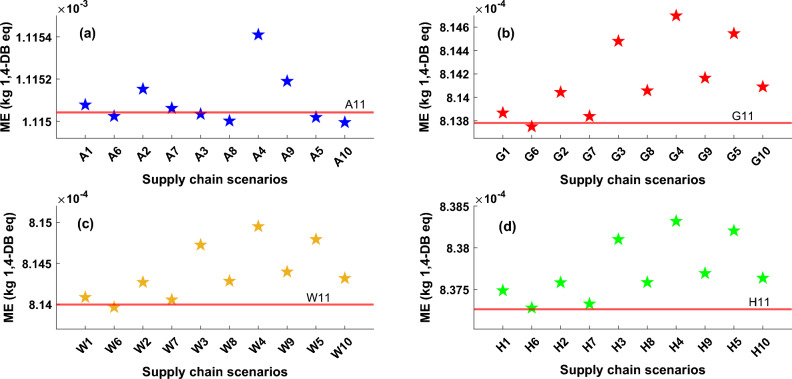
Marine ecotoxicity impact for all heat pump technologies, (**a**) ASHP, (**b**) GSHP, (**c**) WSHP, (**d**) HSHP, The values of marine ecotoxicity impact for all defined scenarios and technologies are presented on these graphs with star markers, red line in each plot represents the impact value based on the UK supply chain scenarios which named A11, G11, W11, and H11 for ASHP, GSHP, WSHP and HSHP types, respectively.

The marine ecotoxicity (ME) category for all technologies and supply chain scenarios is presented in Fig. 4. The manufacturing phase and some applied materials (like steel and copper) in this process cause marine ecotoxicity pollution. The evaluations revealed that the primary factor responsible for the environmental impact of the entire heat pump supply chain is attributed to the extraction of raw materials and manufacturing components. The elevated use of steel and copper during the manufacturing of ASHP results in a greater ME impact for this system in comparison to other technologies. Additionally, in all scenarios, the ME impact of HSHP is marginally higher than that of GSHP and WSHP, primarily because of the increased quantity of these materials required for electrolysis production. Another factor contributing to toxicity is the presence of pollutants in electricity generation networks. Countries with a more environmentally friendly national grid network, such as Sweden, experience fewer toxicity issues in contrast to those with a higher proportion of fossil-fuel-based grids, like China and South Korea. Italy has the highest ecotoxicity impact among considered scenarios for GSHP, WSHP and HSHP, however supposing conducting 50% of the construction process in the UK instead of Italy, results in significant impacts in marine ecotoxicity factor. In the case of the Italy scenario, the increased toxicity impact could be attributed to greater availability of raw materials that contain pollutants, as well as the utilization of more polluting processes. Additionally, a less environmentally friendly source of electricity is employed during the manufacturing process, contributing to this higher toxicity impact. On the other hand, in the supply chain scenario of the UK for GSHP, WSHP, and HSHP, there is a reduced toxicity impact compared to ASHP. Furthermore, minimizing transportation or adopting less pollutant logistical methods during the logistic stage can result in a decreased detrimental impact.

**Figure 5:**
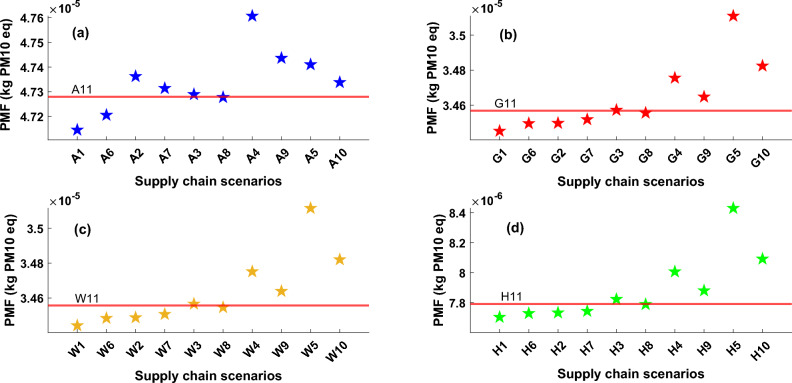
Particulate matter formation impact for all heat pump technologies, (**a**) ASHP, (**b**) GSHP, (**c**) WSHP, (**d**) HSHP, The values of particulate matter formation impact for all defined scenarios and technologies are presented on these graphs with star markers, red line in each plot represents the impact value based on the UK supply chain scenarios which named A11, G11, W11, and H11 for ASHP, GSHP, WSHP and HSHP types, respectively.

Figure 5 displays the particulate matter formation (PMF) impact released by each scenario and technology. PMF within the heat pump supply chain primarily stems from actions linked to raw material extraction, the manufacturing of components, and various industrial processes inherent in heat pump production. These activities have the potential to release particulate matter into the environment, thereby contributing to the overall PMF impact. For ASHP, Sweden has the lowest impact, however, other countries that have higher use of coal and fossil fuel-based power generation have higher damaging consequences. It is evident that considering 50% of heat pump construction in the UK in scenarios A6, G6, W6, and H6 results in a higher impact compared with scenarios that are related to 100% of manufacturing in Sweden (A1, G1, W1, and H1). This is because the manufacturing phase exerts a more significant influence on PMF impact than the other phases within the supply chain. Subsequently, the increased presence of pollutant manufacturing processes in the UK can be attributed to the heightened impact resulting from electricity consumption during the manufacturing phase, serving as the primary factor driving this impact increase. It shows the importance of expanding a greener grid network in the UK to improve the sustainability of the heat pump supply chain. Another significant factor contributing to the impact is the use of materials in the manufacturing of wiring and piping, particularly the utilization of copper, which leads to the generation of PMF impact. In certain impact categories like ME and PMF, HSHP exhibits greater environmental impacts when compared to GSHP and WSHP. This is primarily attributed to the materials used in the manufacturing of the electrolysis, in addition to the refrigerant applied in the heat pump section. This underscores the importance of using innovative and environmentally friendly materials in the construction of hydrogen producer systems to minimize the overall impact during the manufacturing process.

**Figure 6:**
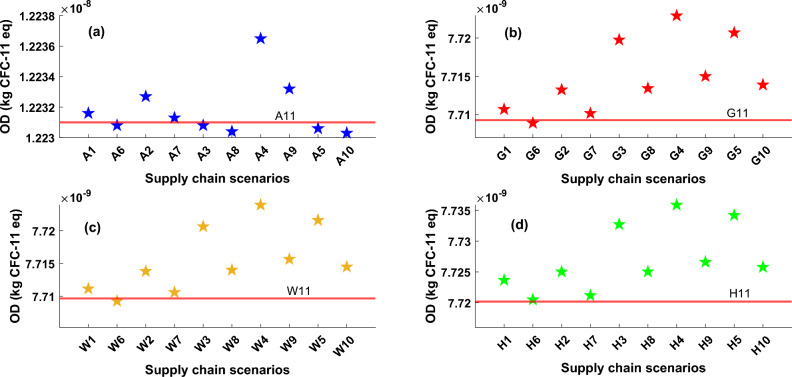
Ozone depletion impact for all heat pump technologies, (**a**) ASHP, (**b**) GSHP, (**c**) WSHP, (**d**) HSHP, The values of ozone depletion impact for all defined scenarios and technologies are presented on these graphs with star markers, red line in each plot represents the impact value based on the UK supply chain scenarios which named A11, G11, W11, and H11 for ASHP, GSHP, WSHP and HSHP types, respectively.

Figure 6 illustrates the ozone depletion (OD) impact category amounts arising from all technologies and scenarios. The main cause of OD is utilizing refrigerants in the manufacturing and operation phases of the heat pump. Refrigerants can be released into the atmosphere, leading to the degradation of ozone molecules in the stratosphere, resulting in the depletion of the ozone layer. It can be seen that the OD impact of ASHP is higher than in other cases due to applying more refrigerant in the manufacturing process of ASHP. Also, energy applied for system operation as well as transportation and distribution part is another contributor to OD-related pollution. For ASHP, scenarios based on manufacturing in South Korea (A2), China (A4), and Sweden (A1) have higher damaging consequences compared with the UK scenario (A11). However, for three other cases, the UK has lower impacts compared with other scenarios which is mainly due to reducing the distribution-related phase reduction. However, Italy has the higher impact which is mainly due to the higher pollutant impact of refrigerant production in Itay and its application in heat pump systems. Also, China and Austria have a high impact on the OD factor. However, it should be mentioned that assuming 50% of the construction of heat pumps in the UK can reduce the OD impact considerably. Also, it is visible that the OD impact of the HSHP supply chain is slightly higher than GSHP and WSHP. Effective management and the reduction of ozone-depleting substances usage are vital steps in addressing and reducing the OD impact within the heat pump supply chain.

**Figure 7:**
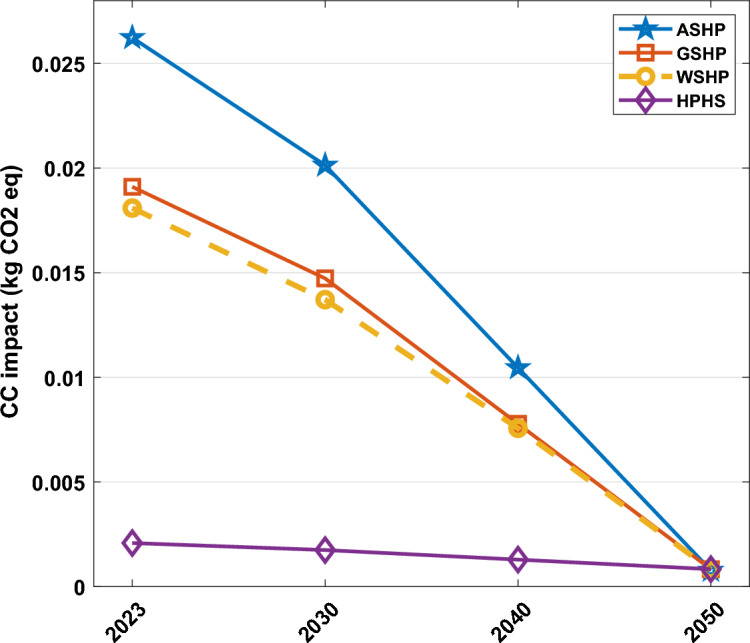
Forecasting analysis of heat pump supply chain environmental impact reduction vs. the UK’s net-zero emission goal, Each line shows the climate change impact variation for a specific technology, the star marker, and blue line are related to ASHP, the square marker, and the red line are related to GSHP, the circle marker, and yellow dash line are related to WSHP, and the diamond marker and purple line are related to HSHP.

**Figure 8:**
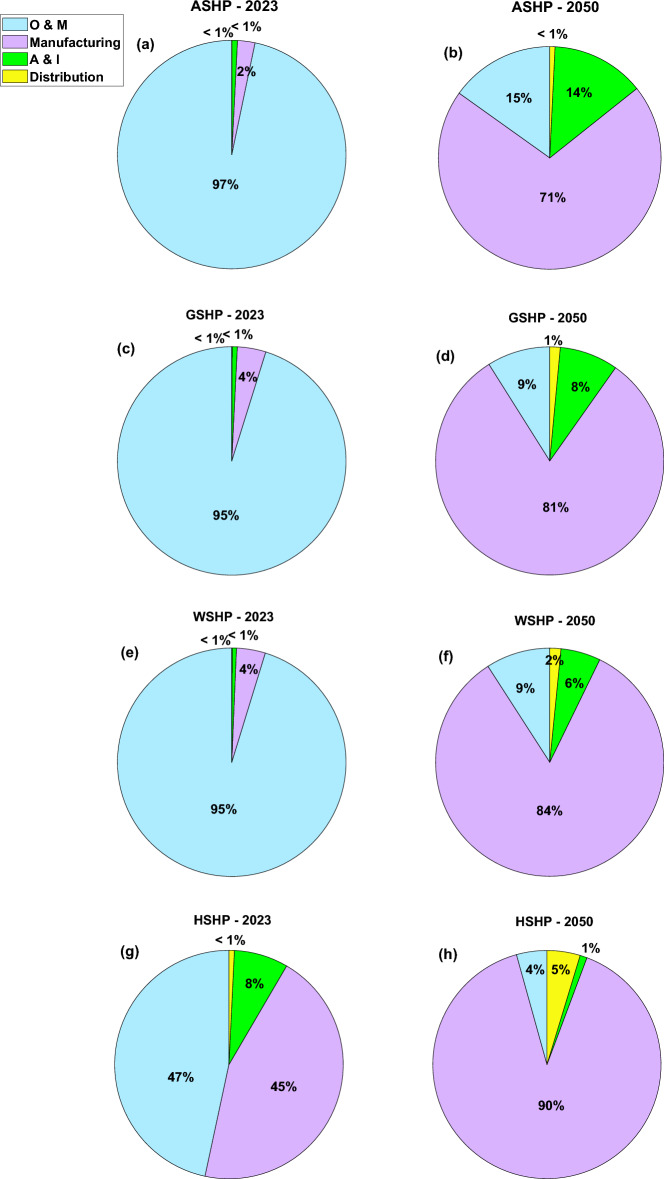
Contribution of each phase to climate change impact for all cases in 2023 vs. 2050, The main portions shown in these pie charts are manufacturing, O&M, A&I, and distribution phases, the figure illustrates the variations of each phase's contribution to damaging impact in 2023 and 2050, (**a**) phases’ portion for ASHP in 2023, (**b**), phases’ portion for ASHP in 2050, (**c**), phases’ portion for GSHP in 2023, (**d**), phases’ portion for GSHP in 2050, (**e**), phases’ portion for WSHP in 2023, (**f**), phases’ portion for WSHP in 2050, (**g**), phases’ portion for HSHP in 2023, (**h**), phases’ portion for HSHP in 2050.

Figure 7 illustrates the carbon footprint reduction of the heat pump supply chain based on the UK’s net-zero emission plan. This graph depicts that the amount of carbon dioxide emissions for ASHP is higher than other technologies and the emissions related to the GSHP and WSHP are similar with a slight difference in 2023. However, the HSHP emissions are remarkably lower than others which is mainly due to less pollution from the operation phase of the heat pump over its lifetime as it has been planned to be supported by green hydrogen, not an electricity grid. All technologies show a decreasing trend from 2023 to 2050 which is UK’s net-zero emission goal. The most significant diminish belongs to ASHP (97% by 2050). It's worth noting that achieving a net-zero emissions plan by 2050 will result in all systems having similar values in terms of carbon footprint criteria. As the heat pump technology has lower emissions compared with gas-fired boilers^42^ which is the main energy provider for the building sector in the UK, investing in the heat pump industry and supply chain with significantly lower environmental impact has been considered as a priority plan for government. One of the main interventions that should be implemented by government and local authorities is promoting and investing in grid network infrastructure in the UK to increase the renewable and clean energies share in the energy mix. Since the O&M phase of heat pumps is the primary source of carbon dioxide emissions and given that existing systems rely on electricity for their operation, a crucial requirement is a more sustainable energy mix. This would be aligned with net-zero emission, energy security, and resiliency plans. On one side, a growing share of renewable resources in the energy mix results in less fossil fuel and natural gas demand, on another side, it eliminates the dependency on natural gas prices and relevant fluctuations as well as availability of the natural gas which will promote the energy security and resiliency in the UK. It also will decrease the environmental impact of the heat pump supply chain which could improve the sustainability of the heat pump industry in the UK. Figure 8 illustrates the various segments within the supply chain contributing to climate change impact in both 2023 and 2050. The findings indicate that, in 2023, a substantial proportion of the adverse impact is linked to the O&M phase. Nevertheless, as renewable energy sources advance by 2050, the environmental impact stemming from the O&M phase of heat pump supply chains diminishes notably across all scenarios. In 2023, the least O&M pollution impact is related to HSHP which is lower than others due to using green hydrogen driven from renewable energies over the lifetime operation of the system. The distribution and A&I phases have a lesser detrimental effect on the overall system lifespan when contrasted with manufacturing and O&M. The primary reason for the impact during the O&M phase is associated with the use of refrigerants, their potential leakage, and replacement throughout the system's life, with a minor impact stemming from component replacements due to breakdowns. The selection of refrigerants can exert a notable influence on global warming. Certain refrigerants possess a high global warming potential (GWP), signifying that they create a potent greenhouse effect when discharged into the atmosphere. Opting for refrigerants characterized by lower GWP values is crucial in mitigating their role in climate change.

## Discussion and conclusion

The challenges of supplying the required heating and cooling demand for the buildings sector in the UK are under government, stakeholders, and local authorities’ attention. Increasing the energy price for households in the UK in recent months, challenges of natural gas application in installed boilers in buildings, its relevant pollution, and dependency on natural gas are the main elements that can threaten the sustainability and energy security and resiliency of building sector in the UK. Expanding different heat pump supply chain scenarios which are the main suppliers of the UK market is done in this study to conduct a comparative LCA of all scenarios. This study seeks to improve the sustainability of the heat pump supply chain. Base scenarios are developed based on 100% of production in exporter countries and then importing them to the UK. Among the evaluated scenarios, Sweden has the most sustainable supply chain path with a lower carbon footprint impact. The computation demonstrates the pivotal importance of the composition of electricity sources in determining the environmental sustainability of the heat pump supply chain. Enhancing the proportion of renewable energy sources within the energy mix can impact a national energy security and resilience. The results indicated that presently, the primary contributor to the carbon footprint of traditional heat pumps is associated with their operation and maintenance (O&M). However, by 2050, as per the defined net-zero emissions plan, the adverse effects of O&M will significantly diminish.

The findings indicated that either setting up a new production line for heat pumps or enlarging the existing one could significantly reduce the environmental footprint. Also, based on the UK government's plan to reach the net-zero emission goal in the building sector, it is crucial to shift to a cleaner system like a heat pump instead of using natural gas-based boilers to improve the energy security in the UK and stably supplying the buildings' energy for peak demands and unpredictable circumstances. However, development of heat pump application and installation should be accompanied by a reduction of environmental impact over the entire supply chain of heat pump. Heat pump supply is immature in the UK and has huge gaps which makes it challenging to develop its application and installation in a short period. Reshoring or production line expansion will reduce the distribution and transportation-related pollution and can guarantee the supply of the UK market demand and decline the dependency on foreign markets.

In the future, once manufacturing capacity is expanded to meet domestic demand, exporting could become advantageous for the UK. These various factors collectively enhance the energy security and supply chain resilience within the heat pump industry. Additionally, this research has explored HSHP (hydrogen-supported heat pumps), which demonstrates significant potential as an eco-friendly option for the building sector. The results have shown that using green hydrogen as an energy source for heat pumps, as opposed to relying on an electricity grid or natural gas in other types of heat pumps and boilers, can substantially reduce the carbon footprint of energy production. For HSHP to thrive, it's not only the heat pump supply chain that needs development and optimization, but also the supply chains for hydrogen production technologies, such as electrolysis, which should be strengthened within the UK. The UK exhibits substantial potential for manufacturing and advancing hydrogen production systems domestically. It's worth noting that the UK government has already established a comprehensive national hydrogen strategy aimed at fostering the production and utilization of hydrogen.

To cover the market with HSHP, both the heat pump and electrolysis supply chain should be developed in parallel unless it can affect the security and resiliency consistency. Another point that should be considered in a resilience heat pump supply chain is manufacturing capacity development to supply all main components of heat pump units in the UK such as compressor, evaporator, valves, piping, insulation, and refrigerants. The UK is well positioned in manufacturing some components like compressor, however, there are considerable gaps in other component supplies.

The findings of this research offer valuable insights to policymakers and stakeholders, providing a comprehensive understanding of the heat pump supply chain throughout its lifecycle. This study has unveiled the environmental consequences associated with each supply chain route. Furthermore, by comparing the options of reshoring and developing heat pump-related industries in the UK and addressing carbon footprint and toxicity concerns^46^, it can assist the government in shaping future trade strategies in alignment with the net-zero emissions plan and a more environmentally sustainable and resilient supply chain strategy. Additionally, this research assessed the supply chain's environmental impact across various timeframes, including projections up to 2030, 2040, and 2050. It showcased the potential of a net-zero emissions plan in mitigating ecological impacts, which can attract investment, garner support from local authorities, and influence decision-makers to allocate more financial resources to expand renewable energy sources and their integration into the grid network. The outcomes of this study could also help policymakers choose more sustainable supply chain scenarios and routes to counter climate change, reduce toxicology and global warming damaging consequences. Such actions to improve UK energy sustainability, resiliency and security are poised to have substantial direct and indirect benefits on a wide range of industries and sectors.

### Supplementary Information

Supplementary Information.

## Data Availability

All data generated or analysed in this study are included in this published article (and its Supplementary Information).
